# Using cognitive interviews with children to test the content validity of a paediatric patient reported experience measure

**DOI:** 10.1136/bmjpo-2026-004589

**Published:** 2026-09-25

**Authors:** Karlen R Barr, Erika Fortunati, Zachary Studniberg, Jessica Nikolovski, Leslie White, Sarah Elliott, Lynn McCartney, Dianna Smith-McCue, Mary Noah, Girish Swaminathan, James R John, Claire Treadgold, Aaron Hall, Ann Dadich, Barb Vernon, Elesha Toscano, Harriet Hiscock, Josephine Chow, Leanne Johnston, Lisa Whitehead, Mandie Foster, Valerie Sung, Valsamma Eapen

**Affiliations:** 1 Academic Unit of Infant Child and Adolescent Psychiatry South Western Sydney Local Health District, Sydney, New South Wales, Australia; 2 Discipline of Psychiatry and Mental Health, University of New South Wales School of Clinical Medicine, Sydney, New South Wales, Australia; 3 Starlight Children’s Foundation, Naremburn, New South Wales, Australia; 4 Ingham Institute for Applied Medical Research, Liverpool, New South Wales, Australia; 5 NHMRC Clinical Trials Centre, University of Sydney, Camperdown, New South Wales, Australia; 6 Faculty of Medicine and Health, Susan Wakil School of Nursing and Midwifery, Sydney Quality of Life Office (SQOLO), University of Sydney, Sydney, New South Wales, Australia; 7 University of New South Wales Faculty of Medicine, Sydney, New South Wales, Australia; 8 Macquarie University Australian Institute of Health Innovation, Sydney, New South Wales, Australia; 9 Women's Healthcare Australasia and Children’s Healthcare Australasia, Mitchell, Australian Capital Territory, Australia; 10 Sydney Children’s Hospitals Network, Sydney, New South Wales, Australia; 11 Women’s and Children’s Health Network, Adelaide, South Australia, Australia; 12 Royal Darwin Hospital, Casuarina, Northern Territory, Australia; 13 Australian Commission on Safety and Quality in Healthcare, Sydney, New South Wales, Australia; 14 Patient Reported Measures Stream, NSW Agency for Clinical Innovation, St Leonards, New South Wales, Australia; 15 School of Business, Western Sydney University, Penrith, New South Wales, Australia; 16 Patient Safety and Quality Service, Children’s Health Queensland, South Brisbane, Queensland, Australia; 17 The University of Melbourne Faculty of Medicine Dentistry and Health Sciences, Melbourne, Victoria, Australia; 18 Children’s Health Queensland Hospital and Health Service, Herston, Queensland, Australia; 19 The University of Queensland Faculty of Health and Behavioural Sciences, Brisbane, Queensland, Australia; 20 School of Nursing and Midwifery, Edith Cowan University, Joondalup, Western Australia, Australia; 21 Health Research Centre, The University of Jordan, Amman, Amman Governorate, Jordan; 22 School of Clinical Sciences, Auckland University of Technology, Auckland, New Zealand; 23 Royal Children’s Hospital, Murdoch Children’s Research Institute The Centre for Community Child Health, Parkville, Victoria, Australia; 24 Department of Paediatrics, University of Melbourne, Melbourne, Victoria, Australia

**Keywords:** Child, Child Health, Patient Rights, Qualitative research

## Abstract

**Background:**

Paediatric patient reported experience measures (pPREMs) can help children’s voices be heard to evaluate and improve healthcare delivery. However, no standardised child-completed pPREM has been co-designed with children and validated in Australia. This research aimed to test the content validity of a co-created pPREM using an iterative, participatory approach with children.

**Methods:**

21 children aged 6–11 years were recruited across four Australian hospitals. Children participated in online cognitive interviews to evaluate pPREM items and rating scales (using ‘stars’ or ‘smiley faces’) for their understandability, usability, relevance and importance related to their needs in hospital. Framework analysis was conducted iteratively, with items refined after the first round of interviews based on children’s feedback.

**Results:**

Most (n=20; 95%) children understood the instructions. After nine interviews, two items were revised: ‘Someone I knew could stay with me’ changed to ‘Family and friends could be with me’ and ‘I was able to talk to other patients’, to ‘I was able to talk to other kids in hospital’. Following these revisions, all children understood most (13 of 18=72%) items. In the remaining five items, some phrases, such as ‘Care and treatment’, were not easily understood, and items were edited according to children’s feedback. Most children (n=14; 67%) preferred the ‘smiley faces’ rating system, and 83% (n=15) reported the number of questions as the right amount. Children generally found all items important, although some items did not apply to all and a ‘Does not apply to me’ response option was added.

**Conclusions:**

Children aged 6–11 years can meaningfully engage with a co-designed pPREM. Cognitive interviews and an iterative approach helped identify and rectify comprehension issues. It is feasible to involve children in the design of pPREMs and doing so strengthens pPREMs’ usability and understandability.

**WHAT IS ALREADY KNOWN ON THIS TOPIC**

- Child-completed paediatric patient reported experience measures (pPREMs) are vital to ensure children can report their hospital experience, in contrast to parent/carer proxy reports.

**WHAT THIS STUDY ADDS**

- This study provides the first evidence in Australia of a co-designed pPREM with validated content, offering a foundation for routine use to capture children’s perspectives in healthcare.
- Children aged 6–11 years actively participated in cognitive interviews that tested the content validity of the pPREM.

**HOW THIS STUDY MIGHT AFFECT RESEARCH, PRACTICE OR POLICY**

- There are substantial gaps in policy and practice regarding the use of pPREMs developed with and for children. This study is a key building block in delivering a pPREM to be used by children in hospital settings.

## Introduction

 Patient reported experience measures (PREMs) are standardised tools to improve the quality and patient-centredness of care.^1–3^ Paediatric PREMs (pPREMs) are used to gather children’s responses regarding healthcare experiences.^4^ pPREMs ensure children’s voices are heard and align with article 12 of the United Nations Convention on the Rights of the Child and the Charter of Children’s and Young People’s Rights in Healthcare Services in Australia.^5 6^ Many pPREMs are completed by a child’s parent/carer or hospital staff member.^4 7^ This can be problematic, as their views can differ from those of children. Furthermore, this approach does not promote the child’s independent right to share their perspective and the values of child-centred care.^7–11^

Children want their hospital experiences to be shared and heard.^11–13^ Therefore, it is important to involve children in pPREM development, although this is rarely achieved.^7 12^ Rigorous qualitative research is recommended to develop items and test PREMs.^14 15^ Demonstrating content validity is also vital and often involves cognitive interviews.^16–19^ Cognitive interviews involve completing a PREM and being asked direct questions to establish what participants are thinking when reading and answering questions.^16 20^ This is particularly important for pPREMs as children might still be developing cognitive and linguistic abilities and might interpret sentences differently to adults.^21^

Existing pPREMs have been developed for adolescents, but few are available for younger children or have been co-designed from the outset. In 2024, a pPREM was developed and validated for young people in Australia aged 12–17 years.^22^ The needs of children questionnaire (NCQ) has also been psychometrically validated in Australia and New Zealand in 2019.^23^ The NCQ used a meta-synthesis of primary research for original item generation, and children provided feedback during the testing stage. For children aged 5–10 years, staff members supported children.^23^ However, a standardised, child-completed pPREM does not exist in Australia for children aged 6–11 years that was co-designed with children from the item generation stage. Current pPREMs have not been validated for children living with disabilities or who are culturally and linguistically diverse.^12 24^ As children living with disabilities and those who are culturally and linguistically diverse both face unique challenges in hospital and experience barriers to sharing their experiences in healthcare systems it is important that pPREM tools are developed and validated with these populations in mind.^25 26^ The current study pre-tested items of a pPREM with children to determine content validity. The aim was to determine whether these items were appropriate and understandable through interviews with children and modify the items as needed.

## Methods

### Study background

A research programme was developed by Children’s Healthcare Australasia, Starlight Children’s Foundation and Sydney Partnership for Health Education Research and Enterprise to develop and validate a pPREM for use in Australian hospitals. Phase 1 involved interviews with children, including children with disabilities and/or who were culturally and linguistically diverse. The interviews explored their hospital experiences and identified what mattered most to them.^13^ Items were then generated using children’s feedback and reviewing existing pPREMs.^4^ An iterative technique using anonymous surveys was completed with expert stakeholders to inform the pPREM tested in this study. Phase 2 was detailed in a study protocol, and the current study focuses on Phase 2a.^27^ The Enhancing the QUAlity and Transparency Of health Research Child Centred Research Checklist and the Consolidated Criteria for Reporting Qualitative Research (COREQ) were used as reporting guidelines for the current study.^11^

### Study setting

To optimise diversity, the study was completed at four hospitals across Australia, in metropolitan, regional and rural areas.

### Participants and recruitment

Children were eligible to participate if they were aged 6–11 years inclusive, and if they had been admitted to a hospital, including an inpatient, outpatient or emergency department admission, over the previous 3 months from the date of consent. Hospital staff members and volunteers provided flyers to children’s family members in hospital. Family members scanned a QR code on the flyer to read study details, confirm eligibility, indicate consent and provide demographic information. Families were provided with time to consider participation and ask questions before consenting. Children provided assent by completing their own simplified assent form online. Participant location was determined via postal code and the modified Monash model.^28^

### Patient and public involvement

JN, a consumer researcher and representative, provided a patient perspective across all study stages. JN contributed to data interpretation and reporting on the findings. Additionally, pPREM items were developed using a co-design process involving interviews with children in Phase 1.^13^

### Data collection

Children participated in online cognitive interviews. They were presented with drafted items and answered questions regarding item clarity.^20^ If the child and parent/carer decided that the parent/carer would be present during the interview, the parent/carer was instructed to allow the child to attempt to respond but they could provide support if needed, such as explaining a word’s meaning. Interviews were conducted via video-conference, and the instructions and items were viewed via screen-sharing. Interpreters were available if needed, although this was not requested by families. KRB, a female research officer with a PhD and 8 years of experience in qualitative research conducted the interviews.

The 18-item pPREM included 15 Likert-scale items and 3 open-ended items. Participants were asked to read each item aloud, explain their understanding and identify parts that were unclear. If children indicated that they were unable to read, the interviewer read the item aloud. pPREM items varied slightly by age group as decided by expert stakeholders in Phase 1 of this project. These variations account for differences in likely developmental capabilities across age groups, with one item specific to 6–8 years old and two specific to 9–11 years old (see table 1).

**Table 1:** Paediatric patient reported experience measures items that were presented to children

Number	Item
1	I understood what my hospital team said to me
2	My hospital team explained things to me before they were done
3	I could ask questions about the care and treatment I was having
4	My hospital team listened to me*
5	My hospital team were friendly and kind
6	I felt safe
7	I was given privacy when I wanted it†
8	I had quiet time when I wanted it*
9	I could choose food I liked from the menu
10	My hospital team helped me if I had pain
11	My hospital team helped me if I was scared*
12	I trusted the hospital team that cared for me†
13	Someone I knew could stay with me when I wanted them to
14	I was able to talk to other patients
15	There were fun things for me to do
16	What was good about your stay in hospital
17	What was not so good about your stay in hospital
18	What else would you like to tell us

* Items specific to 6–8 years old.

† Items specific to 9–11 years old.

First, children were shown the instructions using a ‘faces’ or ‘stars’ rating system and asked whether the instructions were clear (figures 1 and 2). The ‘faces’ or ‘stars’ system was randomly chosen by the interviewer prior to the interview, with approximately half of participants completing the pPREM with either rating system. Participants were then asked to rate an example item ‘I like ice cream’ to see whether they understood the instructions. Next, participants rated the pPREM items and were asked whether items were clear and related follow-up questions (eg, ‘Can you explain how you chose that answer?’, ‘Was anything confusing?’, ‘Are there words that you did not know?’).^16 29^ Probing questions about the unclear parts were posed and participants were asked to suggest improvements. Children were also asked whether they deemed the items important; they preferred a rating system using ‘stars’ or ‘faces’; and the number of items was appropriate. Children were reminded throughout the interview that there were no wrong answers. Interviews were audio-recorded and transcribed verbatim (range=9–39 min, mean=22 min). Families were compensated for their time with a $A40 gift voucher.

**Figure 1:**
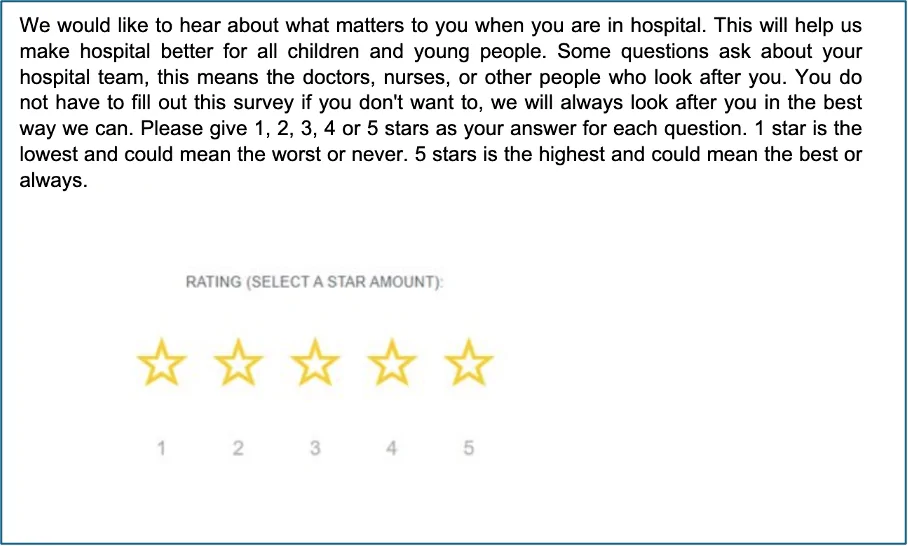
Instructions for ‘stars’ rating system.

**Figure 2:**
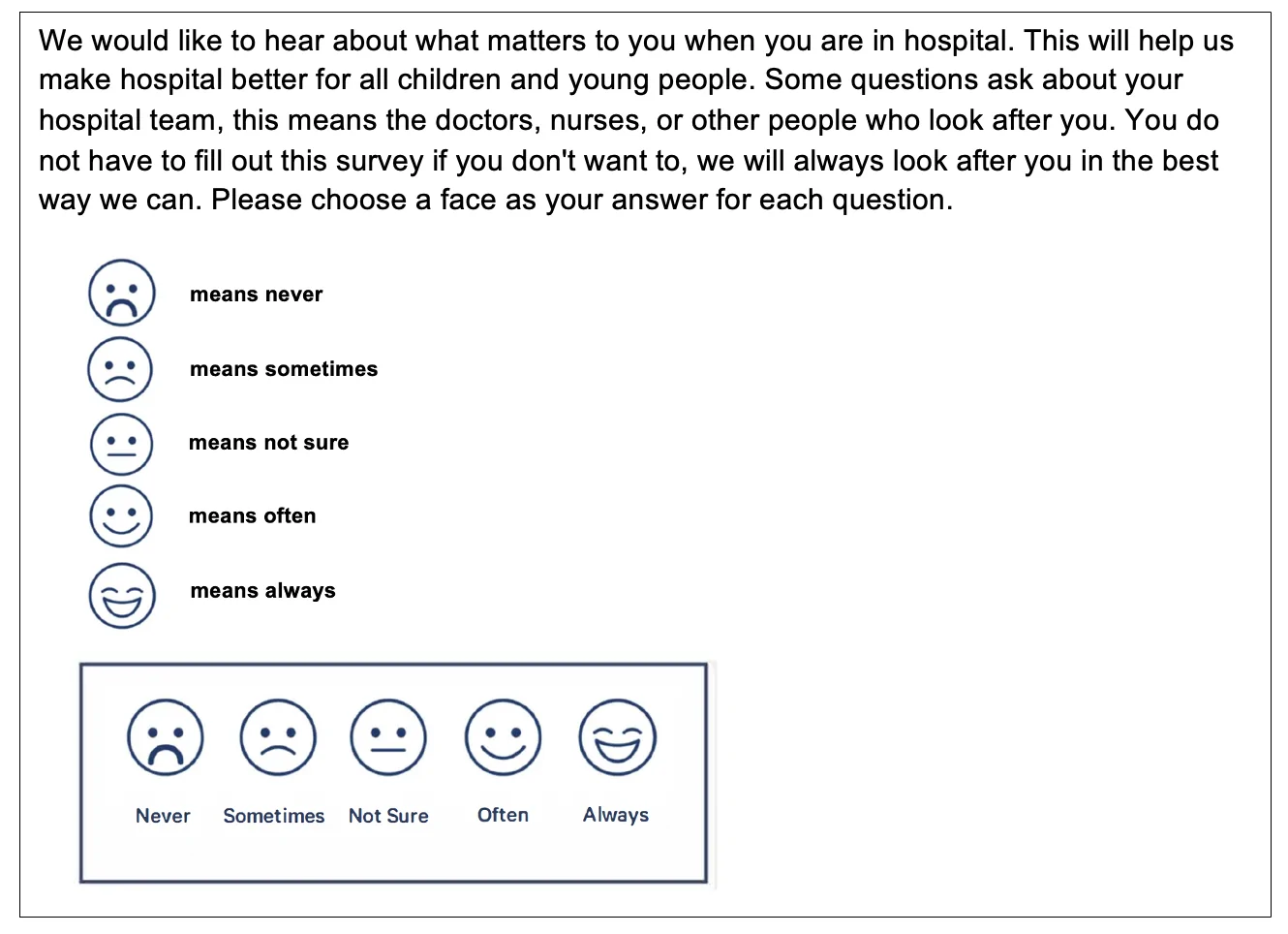
Instructions for ‘faces’ rating system.

Children’s feedback on the pPREM items was considered by the researchers and the Children’s Healthcare Australasia pPREM steering committee. Changes to the items were considered through discussions and an online survey sent to steering committee members. Appropriate changes were made to the items, driven by the children’s responses.

### Data analysis

The analysis of the children’s responses was undertaken by three researchers and aided by NVivo V.12.^30^ An iterative approach was used whereby analysis occurred during data collection, and items were changed in accordance with the child’s feedback. After each third of the total transcripts were analysed (ie, after the 9th and 15th interview), iterative improvements were considered through researcher discussions with the broader study team.

Framework analysis involved: familiarisation with the data by listening to the audio-recordings, re-reading transcripts and documenting notes; independent inductive, line-by-line content coding among the researchers, whereby anything that might be relevant was coded—the researchers then discussed their analysis, and a coding framework was constructed; applying the framework to three transcripts—following this, the researchers discussed and refined the framework; and applying the refined framework to the remaining transcripts.^30^

## Results

### Participant characteristics

21 children participated, an adequate number to attain information power on the pPREM items using cognitive interviews (see table 2).^31 32^

**Table 2:** Demographic characteristics of participants

Mean age, range	8.9, 6–11
6–8 years	8 (38%)
9–11 years	13 (62%)
Gender n (%)	
Female	14 (67)
Male	7 (33)
Aboriginal or Torres Strait Islander n (%)	2 (10)
Main language other than English n (%)	3 (14)
Born overseas n (%)	2 (10)
Identifies as having a disability n (%)	5 (24)
Location n (%)	
Metropolitan	13 (62)
Regional	2 (10)
Rural	6 (29)

### Understanding of pPREM instructions

Most children (n=20; 95%) reported the instructions were clear. One child reported that they did not understand the word ‘survey’ and suggested that ‘questions’ was preferable. Another child suggested a format change by, ‘spac[ing] out the words a bit’ (age=9 years) and another suggested the pPREM could be read aloud to younger children.

### Rating systems can be challenging to understand

Two children indicated they might not have understood the boundaries of one to five for the ‘stars’ rating system as they provided ratings greater than five, including ‘18 stars’ (age=8 years) and ‘10’ (age=6 years). When the rating scale was re-explained, they provided responses between one and five.

Three children tended to respond by describing their experiences or offering commentary rather than using the rating scale. For item 5, one participant said, ‘Yes, it’s really important. So you can feel love while you’re getting the treatment’ (age=9 years). When prompted, they could provide a rating, but often continued explaining their experiences for later items. Additionally, one child did not accurately connect their experience to the ‘stars’ rating system, responding to the example item with, ‘four… I don’t really like ice cream that much’ (age=8 years).

### Rating system and length preferences

67% (n=12) of children preferred the ‘faces’ rating system, and 28% (n=5) preferred the ‘stars’. One child did not have a preference. The ‘faces’ were preferred because they were considered to be fun and visually appealing (eg, cuter), easier to understand and because ‘You get to pick how you feel’ (age=8 years). Children who preferred the stars stated that they made more sense and represented an easier rating system.

Most children (n=15; 83%) advised the number of questions was ‘right’ or a ‘good amount’. One child noted there were too many questions, and two children suggested there were insufficient questions.

### Iterative changes made to pPREM items

After nine interviews, the children’s responses were analysed to determine if changes were needed. Five of nine children had difficulty with item 13, ‘Someone I knew could stay with me when I wanted them to’. Four children noted that, ‘Someone I knew’ referred to doctors and nurses instead of family and friends. Some children found item 13 difficult but could not explain why. Two suggested changing, ‘Someone I knew’ to ‘Family and friends’. Children also noted difficulty with item 14, ‘I was able to talk to other patients’. Two children did not understand the item. Three children suggested changing the word ‘Patient’, and two children reported not understanding the word ‘Patient’. Consequently, item 13 was changed to ‘Family and friends could be with me when I wanted them to’ and item 14, to ‘I was able to talk to other kids in hospital’.

Following 15 interviews, while some children did not understand items 1–3, as well as 6 and 7, it was unclear how the wording should be changed. The interviewer therefore asked additional questions about these in subsequent interviews to better understand and address the confusion.

### Items most understood

All children understood 13 items (4, 5, 8, 9, 10, 11, 12, 13 V.2, 14 V.2, 15, 16, 17 and 18). Children stated they comprehended the item, it was easy, or described an accurate understanding of the item. For example, for item 4, one participant responded, ‘That’s a five. They always listen to me and respected me when I was talking’ (age=9 years).

### Items that were confusing

Some children were confused by certain items. For instance, two 6-year-old children reported that they did not understand the phrase ‘My hospital team’ in item 1. When six children were asked what wording they preferred, three preferred, ‘My hospital team’ and three preferred, ‘Doctors and nurses’.

Similarly, two children reported that item 2 was hard or had ‘too many words’. One child’s response indicated they might have misinterpreted the word ‘done’, stating, ‘They definitely explained it to me after I was done… They explained more to me at the start… [This question was] a little bit harder’ (age=9 years). Two others reported they did not understand the phrase, ‘my hospital team’, although they did not indicate misunderstanding this phrase in item 1. One child acknowledged that, while they understood the item, it might be difficult for others.

Regarding item 3, four children indicated they had difficulty with this, stating they did not understand what care and treatment meant: ‘I’m just not sure what it means about care and treatment’ (age=11 years). Two children suggested replacing the word, ‘treatment’, with ‘medicine’ or ‘surgery’. Alternatives included, ‘I could ask questions about what was going on’ (age=10 years) and ‘I could ask questions about the things that will make me feel better’ (age=10 years).

One child reported they did not understand item 6, stating, ‘it’s in the past tense, so I don’t really know when or what it is or who feels safe’ (age=10 years). Five children who understood the item were asked whether they preferred, ‘I felt safe’ or ‘I felt safe in hospital’ and four of five preferred, ‘I felt safe in hospital’.

One child reported they did not understand the word ‘privacy’ in item 7. Four children who understood this item were asked whether the word ‘privacy’ should be changed, and all four reported preferred keeping the word ‘privacy’.

### Additional difficulties children experienced during interviews

Three children showed boredom, disinterest or limited interest in the interview, with one child requesting, ‘Can you please move on?’ (age=6 years). One 6-year-old child living with a disability was distracted during the interview and reported that he wanted to stop the interview. Three children aged 6–8 years whose first language was English reported difficulty with reading and needed the items read to them. One of these children reported a medical reason for their difficulty. One child found answering item 14 difficult as his friends had died in hospital. Two children found item 16 difficult to answer because they could not think of a response or they did not want to discuss uncomfortable things. One child sometimes explained their parents’ experience: ‘Mum didn’t have quiet time’ (age=8 years). Nine children experienced difficulties with technology during the online interview, such as items appearing small on their phone or unstable internet connection.

### Applicability of items to children

Children sometimes indicated that items were irrelevant to them, including items 2, 7, 9, 10, 14, 17 and 18. For instance, regarding item 2, one child’s mother reported, ‘[He] had severe anxiety from hospital staff… He preferred them not to talk to him’ (mother of 10-year-old child). For item 7, two children indicated that they did not want privacy or that it was difficult to think of a time they needed privacy. Six children described how item 9 did not apply to them because of, for instance, a limited appetite and not being provided a menu: ‘The food was pretty good. My stomach just didn’t have a very big appetite since I was sick’ (age=11 years). Regarding item 10, one child indicated they did not experience pain. Three children reported that item 14 was irrelevant to them or was difficult to answer because they did not want to talk to other children who had been admitted to hospital or they did not talk to them: ‘I never get to talk to other patients… [I didn’t want to] cause I had a tablet,’ (age=8 years).

### Importance of item topics

While the children largely indicated that the items were important to them, some did not share this view. For instance, one child reported that hospital staff members should not be informed of responses to item 1 (age=7 years). The same child reported that items 2 and 3 were not important but could not explain why. One child reported item 5 was not important to ask, ‘because most of the people that work in the hospital are mostly kind… if [children] have a problem, they should talk to others about it’ (age=10 years). The same child noted that item 12 was not important because ‘It’s not really urgent… it’s just a fact’.

### Changes made to the revised pPREM

Several changes were made to the drafted pPREM based on children’s feedback and consultation with the researchers and steering committee. In the instructions, the word ‘questions’ replaced ‘survey’, text was spaced out and the ‘faces’ rating system was chosen over the ‘stars’. Item 1 was unchanged as ‘doctors and nurses’ was deemed non-inclusive of all staff, and parents/carers could explain the concept to children if needed. Item 2 was revised to, ‘My hospital team explained things before they did anything to me’. Item 3 changed to, ‘I could ask questions about what was being done to help me get better’. Item 6 was changed to, ‘I felt safe in hospital’. For item 7, the word ‘privacy’ was retained as many children preferred this. For items that were not applicable to all, a tailored ‘Does not apply to me’ option was added.

## Discussion

This is the first study in Australia to use cognitive interviews with 21 children, aged 6–11 years, to pre-test items of a pPREM to determine content validity. The children understood most items and preferred a ‘faces’ rating system. They were often able to identify what was difficult or confusing about certain items. Iterative changes to items following the first round of interviews resulted in children reporting comprehension of previously confusing items. Children’s feedback was used to revise the pPREM. By incorporating children’s feedback from the outset, this study demonstrates the value of co-designing child-centred PREMs, supporting the meaningful inclusion of children’s voices in evaluating and improving their healthcare experiences.

This study showed that cognitive interviews with children aged 6–11 years helped to identify and address comprehension issues. As per previous research, younger children who were unable to read items or had difficulty explicating their answers, found it difficult to comprehend some items.^21 33^ Younger children might benefit from more concrete cognitive interviewing methods, such as explaining what an item means to a toy.^16 34^ This study demonstrates that children might be able to complete the pPREM independently, although some children might require adult assistance. Children might benefit from choosing who assists them and clear instructions to ensure that children are free to provide their views.^35^

The study findings suggested that the concepts that children deemed important in the first stage of the research continued to be important to them.^13^ For example, children understood and endorsed items 1–5 in this study which corresponded with the first stage of the research where they reported how it was important for them to be involved in shared decision-making, and to have positive relationships with hospital staff. In previous pPREM research, children have described similar aspects of hospital that are important to them, including relationships with hospital staff.^12^ Findings also align with previous research showing children value sharing their hospital experiences.^12^

This study also incorporated the voices of children with diverse backgrounds, including children living with disabilities, children of culturally and linguistically diverse communities and children from regional and rural locations. Generally, the children from diverse backgrounds had similar interview responses to other children, which was also found in the first stage of this study.^13^ However, in the current study, one child living with a disability was distracted and needed to cease the interview.

This study showed the importance of making iterative changes while testing items. Children were able to demonstrate understanding of the two items that were changed after nine interviews. Consultation with children can also help to ensure that tools remain relevant and responsive to their needs and results in improved validity and reliability.^36^

Some children found items in the pPREM uncomfortable or difficult to answer, particularly if they had had a friend die in hospital, aligning with previous research.^37^ Children might benefit from improved consent processes to ensure they understand that answering items in surveys is voluntary and that they can skip questions.^38^ Aligning research practices with child-centred care values may assist to ensure that research is designed to have child participants.^11^ Additionally, the time between children’s hospital experiences and the interview was unclear and might have resulted in recall bias. Research suggests that a delay of several days to a couple of weeks appears to permit accurate recalls and could be a suitable time frame to administer the pPREM.^39^ In response to some items, children occasionally described their experiences rather than using the rating scale. This might have been partly because of the nature of the interview and might not indicate difficulty with the rating system. Children’s tendency to elaborate suggests surveys should clarify response expectations and avoid open-text fields unless intended.

### Implications

By confirming that children as young as 6 years of age can understand and respond to experience measures, this study supports the principle that children’s perspectives should inform quality improvement, rather than relying solely on proxy reports.^12 13 16^ Direct feedback from children can reveal aspects of care that matter most to them, which might be overlooked in adult-centric measures.^12 13^ Incorporating child-reported experience data into hospital systems can help identify gaps in communication, emotional support and environment, leading to more child-friendly care practices.^3 4^ Ultimately, these insights can promote patient-centred care and uphold children’s rights to participate in decisions about their healthcare.^5 6^

### Limitations

The majority of participants were female and 9–11 years and greater representation of females and younger children is needed. While there was some representation of culturally diverse children, children living with a disability and children from rural and regional locations, the numbers were relatively small. During analysis, it was sometimes difficult to verify whether children understood the items and what they were asked.^34^ When children appeared to give patterned responses throughout the interview or simple answers to questions, it could be interpreted as the child not understanding, being disengaged or trying to be socially desirable. However, children might have responded this way for reasons such as being neurodivergent or that they understood and did not wish to elaborate. The online interviews might have been a limitation if children experienced difficulties with reading the items on a small device or where they were less engaged.

### Future research

Future research in our research programme will validate the revised pPREM with a minimum of 180 children, including those from diverse backgrounds, across Australian hospitals.^27^ Research will also explore implementation strategies and integration into routine care.^27^ Future validation should test usability in paper and digital formats without interviewer support. The research programme will result in a freely accessible version of the pPREM to be used in Australian healthcare settings. Future research should validate the pPREM in different healthcare settings, including community and ambulatory settings. Adaptations of the pPREM for children with disabilities and culturally diverse backgrounds need to be further explored and evaluated.^40^ Future research should seek to understand the challenges that children with disabilities and cultural diversity may face when completing the pPREM and what considerations and adjustments can be made for them in practice.^40^ In addition, future pPREM research could investigate whether children elaborate on their responses or whether this was a unique factor due to the interview. This study also demonstrated how iterative co-design processes shaped the pPREM, and future research on the development of psychometric instruments should include iterative processes, such as changing item wording from feedback from end-users.

## Conclusion

This study demonstrates that children aged 6–11 years can meaningfully engage with a pPREM when supported with clear instructions and age-appropriate design. Most children understood the instructions and rated items, confirming the feasibility of direct child reporting in this age group. The findings highlight important considerations for implementation, including the preference for a ‘faces’ rating system. Together, these insights reinforce the importance of iterative, child-centred design to ensure measures are valid and engaging.

## Data Availability

Data are available upon reasonable request.
