# Supplementary material for: Using cognitive interviews with children to test the content validity of a paediatric patient reported experience measure
Source: BMJ Paediatr Open. 2026 Sep 25;10(1):e004589. doi: 10.1136/bmjpo-2026-004589 (PMC13630164; doi:10.1136/bmjpo-2026-004589)
Supplement: Supplementary file 1 [file bmjpo-2026-004589.reviewer_comments.pdf]

PEER REVIEW HISTORY

BMJ Paediatrics Open publishes all reviews undertaken for accepted manuscripts. Reviewers are asked to complete a checklist review form and are provided with free text boxes to elaborate on their assessment. These free text comments are reproduced below.

ARTICLE DETAILS

**TITLE (PROVISIONAL)**	Using cognitive interviews with children to test the content validity of a paediatric patient reported experience measure
**AUTHORS**	Barr, Karlen<br>Fortunati, Erika<br>Studniberg, Zachary<br>Nikolovski, Jessica<br>White, Leslie<br>Elliott, Sarah<br>McCartney, Lynn<br>Smith-McCue, Dianna<br>Noah, Mary<br>Swaminathan, Girish<br>John, James<br>Treadgold, Claire<br>Hall, Aaron<br>Dadich, Ann<br>Vernon, Barb<br>Toscano, Elesha<br>Hiscock, Harriet<br>Chow, Josephine<br>Johnston, Leanne<br>Whitehead, Lisa<br>Foster, Mandie<br>Sung, Valerie<br>Eapen, Valsamma

VERSION 1 – REVIEW

**REVIEWER NAME**	Chiu, Serena
**REVIEWER AFFILIATION**	The University of Sydney
**REVIEWER CONFLICT OF INTEREST**	No Conflict of Interest
**AI DISCLOSURE**	No, I have not used AI
**DATE REVIEW RETURNED**	09-Jun-2026

**GENERAL COMMENTS**	1. "Current pPREMs have not been validated for children living with disabilities or who are culturally and linguistically diverse" The authors may consider describing why developing a standardised pPREM for these populations are important e.g., children living with disability or who are culturally and linguistically diverse experience greater barriers to sharing their experiences in healthcare.<br><br>2. The authors may benefit from using standardised terminology when referencing the participant age group. E.g., the age range is referred to as "aged six to 11 years" throughout discussion but also as "aged 6 to 11 years" throughout other sections of manuscript.<br><br>3. "pPREM items varied slightly by age group." The authors may

	benefit from more explicitly explaining how and why these items varied between age groups.

**REVIEWER NAME**	Small, Jacqueline
**REVIEWER AFFILIATION**	Sydney Local Health District
**REVIEWER CONFLICT OF INTEREST**	No Conflict of Interest
**AI DISCLOSURE**	No, I have not used AI
**DATE REVIEW RETURNED**	06-Jul-2026

**GENERAL COMMENTS**	Excellent and timely research. Well presented paper with important outcomes for engaging children and young people in their healthcare

VERSION 1 – AUTHOR RESPONSE

Reviewer: 1
1. "Current pPREMs have not been validated for children living with disabilities or who are culturally and linguistically diverse" The authors may consider describing why developing a standardised pPREM for these populations are important e.g., children living with disability or who are culturally and linguistically diverse experience greater barriers to sharing their experiences in healthcare.

The following sentence has been added: As children living with disabilities and those who are culturally and linguistically diverse both face unique challenges in hospital and experience barriers to sharing their experiences in healthcare systems it is important that pPREM tools are developed and validated with these populations in mind [26-32].

2. The authors may benefit from using standardised terminology when referencing the participant age group. E.g., the age range is referred to as "aged six to 11 years" throughout discussion but also as "aged 6 to 11 years" throughout other sections of manuscript.

Terminology has been standardised to “six to eleven years” throughout the manuscript

3. "pPREM items varied slightly by age group." The authors may benefit from more explicitly explaining how and why these items varied between age groups.

The following explanation has been added: These variations account for differences in likely developmental capabilities across age groups, with one item specific to six to eight year-olds and two specific to nine to eleven year-olds (see Table 1).

Reviewer: 2
Excellent and timely research. Well presented paper with important outcomes for engaging children and young people in their healthcare

VERSION 2 – REVIEW

**REVIEWER NAME**	Chiu, Serena
**REVIEWER AFFILIATION**	The University of Sydney
**REVIEWER CONFLICT OF INTEREST**	No Conflict of Interest
**AI DISCLOSURE**	No, I have not used AI
**DATE REVIEW RETURNED**	25-Aug-2026

**GENERAL COMMENTS**	No further comments.

**REVIEWER NAME**	Small, Jacqueline
**REVIEWER AFFILIATION**	Sydney Local Health District
**REVIEWER CONFLICT OF INTEREST**	No Conflict of Interest
**AI DISCLOSURE**	No, I have not used AI
**DATE REVIEW RETURNED**	03-Aug-2026

**GENERAL COMMENTS**	Important paper
